# Efficacy and Acceptability of Orthokeratology for Slowing Myopic Progression in Children: A Systematic Review and Meta-Analysis

**DOI:** 10.1155/2015/360806

**Published:** 2015-06-11

**Authors:** Daizong Wen, Jinhai Huang, Hao Chen, Fangjun Bao, Giacomo Savini, Antonio Calossi, Haisi Chen, Xuexi Li, Qinmei Wang

**Affiliations:** ^1^School of Ophthalmology and Optometry, Wenzhou Medical University, 270 West Xueyuan Road, Wenzhou, Zhejiang 325027, China; ^2^Department of Ophthalmology, No. 180 Hospital of Chinese PLA, Quanzhou, Fujian, China; ^3^Key Laboratory of Vision Science, Ministry of Health P.R. China, 270 West Xueyuan Road, Wenzhou, Zhejiang 325027, China; ^4^G.B. Bietti Foundation IRCCS, Rome, Italy; ^5^Department of Physics (Optics and Optometry), University of Florence, Florence, Italy

## Abstract

*Background*. To evaluate the efficacy and acceptability of orthokeratology for slowing myopic progression in children with a well conducted evidence-based analysis.* Design*. Meta-analysis.* Participants*. Children from previously reported comparative studies were treated by orthokeratology versus control.* Methods*. A systematic literature retrieval was conducted in MEDLINE, EMBASE, Cochrane Library, World Health Organization International Clinical Trials Registry Platform, and ClinicalTrials.gov. The included studies were subjected to meta-analysis using Stata version 10.1.* Main Outcome Measures*. Axial length change (efficacy) and dropout rates (acceptability) during 2-year follow-up.* Results*. Eight studies involving 769 subjects were included. At 2-year follow-up, a statistically significant difference was observed in axial length change between the orthokeratology and control groups, with a weighted mean difference (WMD) of −0.25 mm (95% CI, −0.30 to −0.21). The pooled myopic control rate declined with time, with 55, 51, 51, and 41% obtained after 6, 12, 18, and 24 months of treatment, respectively. No statistically significant difference was obtained for dropout rates between the orthokeratology and control groups at 2-year follow-up (OR, 0.79; 95% CI, 0.52 to 1.22).* Conclusions*. Orthokeratology is effective and acceptable for slowing myopic progression in children with careful education and monitoring.

## 1. Introduction

Myopia has emerged as a worldwide public health issue [1] because of its dramatically increased prevalence during the past few decades, especially in East Asia [2–5], with 60–90% youngsters affected. Myopia occurs at a relatively young age and is more likely to progress to high myopia. In turn, high myopia is associated with a high risk of blinding complications such as myopic fundus disease, cataract, and glaucoma [6, 7]. All the abovementioned ailments require significant health care expenditure and are related to declined vision-related quality of life [8].

Myopic progression in young children is primarily due to eye elongation; in other words, the elongation of the axial length (AL) must be slowed to control myopia [9, 10]. For decades, researchers and clinicians have proposed approaches to slow myopic progression [11]. However, no ideal therapeutic pathway has been identified for effective myopic progression reduction or prevention when efficacy, safety, economic feasibility, and clinical acceptability are fully considered.

Orthokeratology (OK) was first introduced in the early 1960s and is based on gas permeable contact lenses that temporarily reshape the cornea surface to reduce myopia progression [12]. However, OK is not widely used for safety concerns and outcome unpredictability [13–15]. Interestingly, previous studies have reported that axial length elongation in children wearing OK lenses is reduced by 32–55% compared with their counterparts wearing single-vision spectacles or soft contact lenses [16–23]. However, these studies differed in baseline refraction, baseline age, axial length measurements, ethnicities, and type of treatment and control, with some limited by small sample sizes. It therefore is necessary to evaluate the efficacy and acceptability of OK for slowing myopic progression in children with a well-conducted evidence-based analysis.

In the current study, a systematic review, with meta-analysis of existing high-quality evidence, was carried out, aiming to provide a robust estimate of the efficacy and acceptability of OK for slowing myopic progression in children.

## 2. Materials and Methods

### 2.1. Search Strategy

Studies describing the comparative outcomes of orthokeratology and controls were identified through a systematic literature search (up to November 1, 2014) in MEDLINE, EMBASE, and the Cochrane Library. Ongoing trials were retrieved by searching the World Health Organization International Clinical Trials Registry Platform and clinicaltrials.gov. The following search strategy was performed in PubMed: (((short OR near∗) AND sight∗) OR myop∗ OR myopia [MeSH]) AND “Orthokeratologic Procedures” [MeSH] OR orthokeratolog∗ OR (corneal AND reshap∗) AND (Refractive Errors [MeSH] OR refract∗ OR Accommodation, Ocular [MeSH] OR Visual Acuity [MeSH] OR (accommodat∗ or acuity) OR (progress∗ or slow∗ or retard∗ or funct∗)). Language restrictions were not used. The titles and abstracts of all identified articles meeting the present study objective were screened for selection firstly. The full-text articles of included trials were collected and assessed according to our preset inclusion criteria. Reference lists of the included articles were searched to find additional information about relevant studies.

### 2.2. Inclusion and Exclusion Criteria

The following inclusion criteria were adopted: (i) articles reporting randomized controlled studies (RCTs) and cohort studies; (ii) for study population, patients aged 6–15 years with any degree of myopia, no significant copathology, no history of ocular surgeries, or no systemic disease associated with impaired or abnormal wound healing; (iii) as intervention, eyes treated by orthokeratology versus control.

For the paucity of relevant trials, the search was not limited to RCTs, and nonrandomized clinical trials, including prospective and retrospective cohort studies, were also included. Review articles, animal or laboratory studies, letters, and conference abstracts were excluded.

### 2.3. Outcome Measures

Axial length change and dropout rates during the 2-year follow-up were assessed as the primary outcomes. The mean axial length change and myopia control rate (treatment slowed axial elongation compared to control) during the treatment were used to assess efficacy. The number of patients who terminated the study early for any reason during the treatment (dropout rate) determined the acceptability.

### 2.4. Data Extraction

Two authors (Daizong Wen and Jinhai Huang) working independently extracted the data and performed the methodological quality assessment of the included studies. The following information was recorded for each eligible trial: authors' names, year of publication, study design, ethnicity of participants, duration of follow-up, instrument for axial length measurements, type of treatment and control, number of subjects, initial mean spherical equivalent refraction, and initial mean axial length. Mean axial length change and dropout rates were also recorded.

### 2.5. Qualitative Assessment

The quality of the trials was assessed using the Jadad scale for randomized, controlled studies and the Newcastle-Ottawa Scale (NOS) for nonrandomized cohort studies [25, 26]. Three main areas of assessment including randomization, blinding, and participant withdrawals/dropouts were contained in the Jadad scale, whose total score ranged between 0 and 5. Studies scoring more than 3 points were considered of high quality. The NOS scores ranged from 0 to 9 points for the quality of selection, comparability, and outcome measures. A score >6 points was considered of high quality.

### 2.6. Statistical Analysis

Outcome data were analyzed by Stata version 10.1 (StataCorp, LP, College Station, TX) using the metan program. In the meta-analysis, weighted mean difference (WMD), odds ratios (ORs), and ninety-five percent confidence intervals (CIs) were calculated for continuous outcomes, dichotomous outcomes, and summary estimates, respectively. A *p* value of less than 0.05 was considered statistically significant. The chi-square and *I* ^2^ statistics were determined for assessment of heterogeneity between studies. The definition of *I* ^2^ is bounds for low (<25%), moderate (~50%), and high (>75%) heterogeneity [27]. In case of significant heterogeneity (*I* ^2^ > 50%) or clinical diversity, the Mantel-Haenszel method for random-effects model was used to pool the data. A random-effects meta-analysis was also performed by the DerSimonian-Laird method if significant heterogeneity was observed. The myopia control rate was calculated using the following formula: myopia control rate = |mean  axial  length  change  in  orthokeratology − mean  axial  length  change  in  control|/mean axial length change in control.

Sensitivity and subgroup analysis was performed by evaluating the effects of study design (randomized or nonrandomized, prospective, or nested), ethnicity (Asian or Caucasian), measurement instrument (A-Scan or IOLMaster), type of treatment and control (OK versus single vision spectacles, OK versus soft contact lenses, partial reduction orthokeratology versus single vision spectacles, or toric orthokeratology versus single vision spectacles), and treatment duration (6, 12, 18, and 24 months) on outcomes to detect differences between groups and determine the sufficiency and strength of the findings that lead to conclusions.

## 3. Results

**Figure 1:**
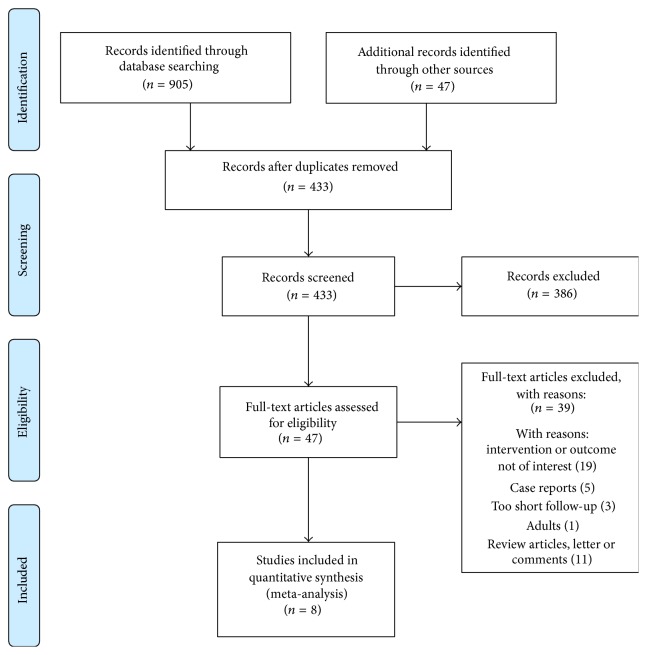
Results of literature search strategy.

A total of 433 reports were identified by the literature search. Based on title and abstract review, 386 articles were ruled out for their obvious irrelevance. In addition, 39 studies that did not meet our inclusion criteria were excluded from further full paper review (Figure 1). Finally, eight studies describing a total of 769 subjects with myopia ranging from 0 to −10.00 D (382 assigned to the treatment group and 387 to the control group) were included in this meta-analysis [16–23]. Two studies were randomized controlled trials (RCTs) [19, 22], and the remaining 6 were cohort trials [16–18, 20, 21, 23]. Six of the eight studies used common OK as treatment [16–21], while the remaining two used partial reduction orthokeratology (PRok) and toric orthokeratology (Tok), respectively [22, 23]. In the control group, seven studies used single vision spectacles (SV) [16, 18–23] and one employed soft contact (SC) lenses [17]. The characteristics and quality assessment of the included studies are summarized in Tables 1 [Table tab2] [Table tab3]–4. The good quality of the studies was confirmed according to the quality scoring described before: the two RCT studies scored 4 points on the Jadad scale and the six nonrandomized cohort studies had 6–9 points on the NOS.

### 3.1. Efficacy

**Figure 2:**
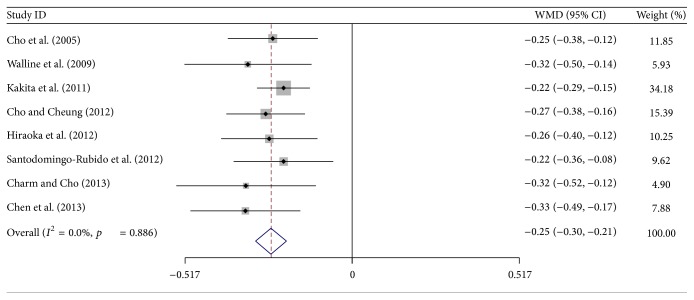
Mean difference of axial length change between orthokeratology and control at 2-year follow-up. WMD = weighted mean difference.

Figure 2 shows the pooled weighted mean difference (WMD) of axial length change between orthokeratology and control at 2-year follow-up: a statistically significant difference was found (WMD, −0.25 mm; 95% CI, −0.30 to −0.21). No significant heterogeneity was detected (*I* ^2^ = 0%), and a fixed-effects model was used. The pooled myopic control rate was 41%.

### 3.2. Acceptability

**Figure 3:**
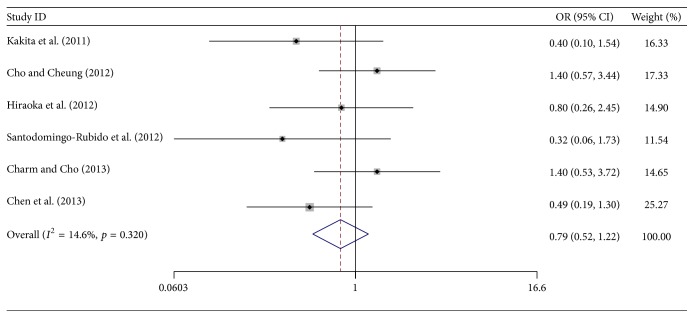
Odds ratios (OR) of dropout rates between orthokeratology and controls at 2-year follow-up.

Figure 3 shows the pooled ORs of dropout rate between orthokeratology and control at 2-year follow-up. No significant difference was obtained after statistical analysis (OR, 0.79; 95% CI, 0.52 to 1.22). Here also, no significant heterogeneity was obtained (*I* ^2^ = 14.6%), and a fixed-effects model was used.

### 3.3. Sensitivity and Subgroup Analysis

#### 3.3.1. Different Types of Study Design

When calculated with different types of study design (randomized or nonrandomized, prospective, or nested), the original findings on efficacy were not significantly influenced (Table 5). However, nonrandomized trials demonstrated a statistically significant difference in dropout rate/acceptability (OR, 0.51; 95% CI, 0.28 to 0.92).

#### 3.3.2. Different Ethnicities

The analyzed data showed no statistically significant difference between Asian and Caucasian children for both efficacy and acceptability, and the results did not substantially contradict the original findings between orthokeratology and control (Table 5).

#### 3.3.3. Different Instruments of Measurement

The statistical results for efficacy and acceptability did not substantially change the original findings of the comparison between orthokeratology and control, in both A-Scan and IOLMaster groups, and no significant difference in efficacy was detected between them (Table 5).

#### 3.3.4. Different Types of Treatment and Control

Although statistical analysis of each subgroup demonstrated the robustness of the original results for both efficacy and acceptability (Table 5), the effect size of OK versus SC (WMD, −0.32 mm; 95% CI, −0.50 to −0.14), PRok versus SV (WMD, −0.32 mm; 95% CI, −0.52 to −0.12), and Tok versus SV (WMD, −0.33 mm; 95% CI, −0.49 to −0.18) in efficacy was slightly stronger than that of OK versus SV (WMD, −0.24 mm; 95% CI, −0.29 to −0.19).

#### 3.3.5. Different Treatment Durations

**Figure 4:**
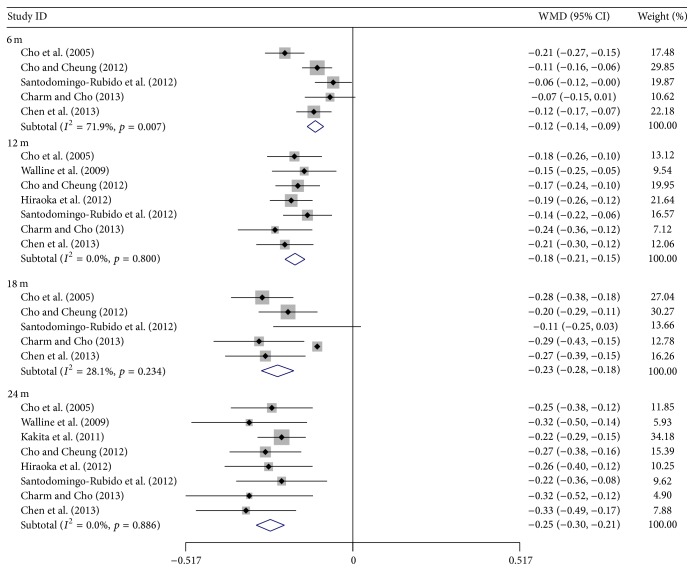
Mean differences of axial length change between orthokeratology and control with different treatment durations. WMD = weighted mean difference.

**Figure 5:**
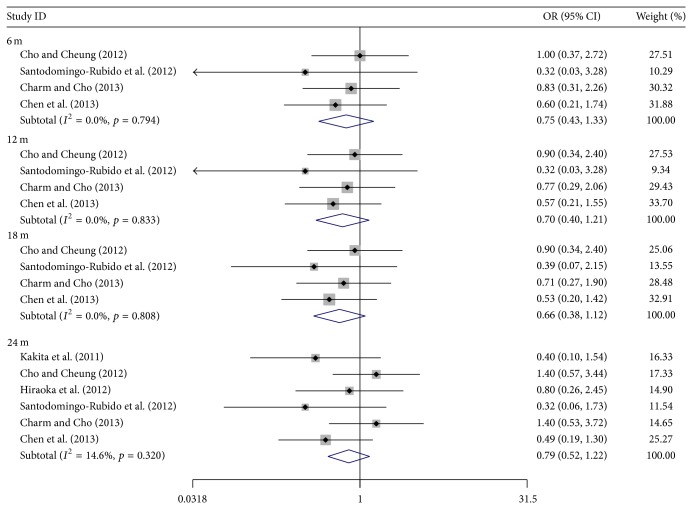
Odds ratios (OR) of dropout rates between orthokeratology and controls with different treatment durations.

Figures 4 and 5 show the pooled estimates of the treatment efficacy and acceptability with different treatment durations. The WMD of AL change between treatment and control increased gradually with time, while the myopia control rate declined, with 55, 51, 51, and 41% recorded for 6, 12, 18, and 24 months, respectively (Table 6). In acceptability, no statistically significant difference of the dropout rate was found in any of the four treatment durations between the two groups.

## 4. Discussion

The purpose of the current meta-analysis was to summarize the available relevant evidence and address the efficacy and acceptability of OK in myopic children. Our findings supported the previous reports [16–23] that OK can efficiently slow axial elongation in myopic children: the myopia control rate was 41% compared to control at 2-year follow-up; the mean difference of axial length change between OK and control was 0.25 mm, which was 0.12 mm in pirenzepine study and 0.40 mm in atropine study [28, 29]. In addition, the statistics of the dropout rate showed that OK's acceptability seemed to be no worse than that of control.

We included not only RCTs but also nonrandomized cohort studies in the present meta-analysis; this may result in potential bias and overestimation of treatment effects [30]. However, previous findings have demonstrated that the results of well-designed and high-quality observational studies were dramatically similar to those of RCTs [31], and such nonrandomized trials may make up for the ethical problems of RCTs [32]. Enrolling of nonrandomized observational studies has also been done in previous meta-analyses [33–36]. For the observational studies included in this analysis, NOS generated scores of 6–9, which indicates high quality. Moreover, our sensitivity analyses of different types of study designs (randomized or nonrandomized, prospective, or nested) showed that WMD results were comparatively stable, indicating that study design may not be a significant factor influencing efficacy. However, in the nonrandomized group, the dropout rate in OK was significantly lower than that in control, while no significant difference was found in the randomized group. The reason may be that the subjects selected in the OK group are those willing to wear OK in nonrandomized studies, and it was easier for them to stick with the OK lens.

Orthokeratology flattens the central cornea while steeping midperipheral to reduce relative peripheral hyperopia, which would slow the rate of myopia [37, 38]. The degree of relative peripheral hyperopia in East Asians is higher in comparison with Caucasians, and myopia progression in East Asian children is generally significantly more pronounced than in children from Western countries [39, 40]. Moreover, reading habits, near work, outdoor activities, and other environmental aspects differ among various ethnicities [41–43]. Hence, the myopia progression rates seem to be affected by the variation in ethnicity between studies. Interestingly, a previous meta-analysis of myopia control with Multifocal Lenses (MLs) versus Single Vision Lenses demonstrated that Asian children appeared to benefit more from MLs than white children. However, our study with OK showed no significant difference between these ethnic groups. Of note, only two papers containing Caucasians were included in our study and the control groups were different in those trials. Therefore our results and conclusions might be affected. More studies and articles are anticipated for a more convincing conclusion.

The accuracy of axial length measurement plays an extremely important role in the observation of OK effect on controlling myopia. In previous studies, axial length measurements were performed with an ultrasonic A-mode device in orthokeratology subjects [16, 17]. But this classical contact-type measurement may be difficult to use in assessing axial length precisely with proper fixation and no compressive stress in children [44]. Therefore, recent studies [18–23] evaluated the axial length using a noncontact optic biometric device (IOLMaster; Carl Zeiss Meditec), which is more suitable for children: high reproducibility, precision, noncontact, and velocity. In the current study, the results of the subgroup analysis between these two devices showed no significant difference. So, different devices do not result in obvious deviation when measuring the axial length during treatment with OK, although the IOLMaster seems to be more appropriate for children.

Differences in type of treatment and control were found in the studies included in the current meta-analysis; this may result in heterogeneity among studies. Therefore, sensitivity and subgroup analyses were conducted to determine the impact of the diversity. Statistical analysis showed slightly stronger effect size of OK versus SC (Walline et al. study [17]), PRok versus SV (Charm and Cho study [22]), and Tok versus SV (Chen et al. study [23]), compared with OK versus SV (pooled estimates of other 5 studies) in axial length control. However, a difference in axial length with a value within 0.1 mm in two years is small in terms of clinical significance. In Walline et al.'s study [17], soft contact lens was used in the control group. As soft contact lenses do not increase myopic progression compared with spectacles [45, 46], they believed that soft contact lens wearers are appropriate controls. However, their control groups were recruited from different historical studies; this may lead to latent bias and inflate the treatment effect. Charm and Cho's study [22] used PRok to slow myopic progression in high myopia patients with spherical equivalent refraction of at least −5.75 diopters. They suggested that greater midperipheral corneal changes in these subjects may result in better control effect than low to moderate myopic subjects in other studies. Chen et al.'s study [23] used Tok to control myopia in children with moderate to high astigmatism with a mean age of just 9.15 years, the minimal in all the included studies. Previous studies have confirmed that OK treatment would be more beneficial to younger than older myopic children [19, 20]. This may be the reason why better control of myopic progression was shown in Chen et al.'s study.

ROMIO study [19] reported a time dependent apparent reduced efficacy on myopic control using OK, which was also observed in Hiraoka et al.'s results [20]. In their opinion, the reduction resulted from the gradual slowing of myopic progression in the control group with age, which was confirmed as a natural process, instead of reduced OK efficacy [47–49]. The results of our meta-analysis also proved that myopic progression in control group slowed with time, and myopic control rate decreased from 51% in the first 6 months to 41% by the end of the study. Therefore the apparent reduction in axial elongation in control subjects may have neutralized the efficacy of OK, lessening the differences between the two groups, and thus giving the impression of decreased efficacy of myopic control treatment with time.

The safety of OK use is always a concern for clinicians and researchers.  Table 7 summarizes the adverse events of included studies; it was encouraging to find that, with careful education and monitoring to both parents and children during the course of the treatment, the complications associated with OK lens wearing can be minimized. Indeed, no severe adverse events leaving permanent damage to the eye or vision during the treatment period were reported in the included studies. The most common complication reported was corneal staining, but in most cases it was slight and could be monitored and easily managed. But severe complications in young wearers such as microbial keratitis had been reported in some observational studies and case studies when wearing OK lens [24, 50, 51]. Therefore, specific education and regular eye examination are quite essential to ensure efficacy and safety.

To be considered an optimum method for treatment, OK should be developed with good acceptability besides demonstrated efficacy and safety, while providing convenience for children's daily activities. In the current study, we selected dropout rates as observation index for acceptability. High dropout rates mean that the treatment would not be useful. The dropout rates in the OK group ranged from 7% [18] to 54% [22] at 2-year follow-up in the included studies. Charm and Cho's study [22] reported an apparently high dropout rate (54%) in the OK Group but also found a high dropout rate (38%) in control patients wearing spectacles. The authors suggested the main reason not to be nonacceptability and attributed the high dropout rate to the relatively small sample size used. Our current meta-analysis of acceptability (dropout rate) demonstrated that there was no significant difference between OK and control at 2-year follow-up, which supports the acceptability of OK as treatment for myopic children.

There are several limitations to our study. First, the sample size of included studies was relatively small, and this may lead to deviation or failure to detect actual differences. Second, some considerable parameters, including age and the dioptric refractive error, which might influence myopic control by OK, were not analyzed, due to the lack of data. Third, except one study with 5-year follow-up, the other studies only lasted 2 years. The long-term efficacy and acceptability of OK, as well as the rebound phenomenon after discontinuation of contact lens use, need more long-term researches to confirm our data.

In conclusion, with careful education and observation, OK lens use is effective and acceptable for slowing myopic progression in children. Moreover, as the efficacy on myopic control by OK lenses reduces with increasing age, early intervention with OK in young children may be worth considering to reduce the prevalence of high myopia. Further well-organized, randomized, and prospective studies with larger sample size and longer follow-up periods are required to confirm the findings described herein.

## Figures and Tables

**Table 1:** Characteristics of included trials.

Study	Design	Year	Ethnicity	Follow-up (years)	Measurements of AL	Treatment group					Control group				
						Type	Subjects	Age	Initial SER	Initial AL	Type	Subjects	Age	Initial SER	Initial AL
							(*n*)	(years)	(D)	(mm)		(*n*)	(years)	(D)	(mm)
Cho et al. [16]	CT (Nested)	2005	Chinese	2	A-Scan	Ok	43	9.57 ± 1.46	−2.27 ± 1.09	24.50 ± 0.71	SV	35	9.58 ± 0.69	−2.55 ± 0.98	24.64 ± 0.58

Walline et al. [17]	CT (Nested)	2009	Caucasian	2	A-Scan	Ok	40	10.5 ± 1.1	NR	24.30 ± 0.73	SC	28	10.5 ± 1.0	NR	24.20 ± 0.63

Kakita et al. [18]	CT (Prospective)	2011	Japanese	2	IOLMaster	Ok	90	12.0 ± 2.6	−2.55 ± 1.82	24.66 ± 1.11	SV	120	11.9 ± 2.1	−2.59 ± 1.66	24.79 ± 0.80

Cho and Cheung [19]	RCT (Prospective)	2012	Chinese	2	IOLMaster	Ok	51	9.23 ± 1.06	−2.05 ± 0.72	24.48 ± 0.71	SV	51	9.39 ± 1.00	−2.23 ± 0.84	24.40 ± 0.84

Hiraoka et al. [20]	CT (Prospective)	2012	Japanese	5	IOLMaster	Ok	58	10.04 ± 1.43	−1.89 ± 0.82	24.09 ± 0.77	SV	60	9.95 ± 1.59	−1.83 ± 1.06	24.22 ± 0.71

Santodomingo-Rubido et al. [21, 24]	CT (Prospective)	2012	Caucasian	2	IOLMaster	Ok	31	9.6 ± 1.6	−2.20 ± 1.09	24.49 ± 0.78	SV	30	9.9 ± 1.9	−2.35 ± 1.17	24.26 ± 1.01

Charm and Cho [22]	RCT (Prospective)	2013	Chinese	2	IOLMaster	PRok	26	10	−6.41	26.02 ± 0.57	SV	26	10	−6.22	25.93 ± 0.54

Chen et al. [23]	CT (Prospective)	2013	Chinese	2	IOLMaster	Tok	43	9.4 ± 1.4	−2.46 ± 1.32	24.37 ± 0.88	SV	37	8.9 ± 1.6	−2.04 ± 1.09	24.18 ± 1.00

RCT = randomized controlled trial; CT = cohort trial; SER = spherical equivalent refraction; AL = axial length; Ok = orthokeratology contact lenses; PRok = partial reduction orthokeratology; Tok = toric orthokeratology; SV = single vision spectacles; SC = soft contact lenses; NR = not report.

**Table 2 tab2:** Efficacy (mean AL change) and acceptability (dropout rate) in each study at 2-year follow-up.

	Mean AL change (mm)		Dropout rate (dropouts/total)	
	Treatment group	Control group	Treatment group	Control group
Cho et al. [16]	0.29 ± 0.27	0.54 ± 0.27	8/43	NA
Walline et al. [17]	0.25 ± 0.27^a^	0.57 ± 0.40^a^	12/40	NA
Kakita et al. [18]	0.39 ± 0.27	0.61 ± 0.24	3/45	10/60
Cho and Cheung [19]	0.36 ± 0.24	0.63 ± 0.26	14/51	10/51
Hiraoka et al. [20]	0.45 ± 0.21	0.71 ± 0.40	7/29	9/30
Santodomingo-Rubido et al. [21, 24]	0.47 ± 0.20^b^	0.69 ± 0.30^b^	2/31	6/30
Charm and Cho [22]	0.19 ± 0.21	0.51 ± 0.32	14/26	10/26
Chen et al. [23]	0.31 ± 0.27	0.64 ± 0.31	8/43	14/37

AL = axial length; NA = not available.

a used the highest sd value of other studies with the same follow-up period.

b derived from standard error in combination with GetData Graph Digitizer 2.24 (http://getdata-graph-digitizer.com/).

**Table 3 tab3:** Jadad scale for randomized controlled studies.

Study	Randomization	Blinding	Withdrawals	Sum of score
Cho et al. [16]	●●	●	●	4
Charm and Cho [22]	●●	●	●	4

Jadad scale allocates 1 point for the presence of each of the following: randomization, blinding, and participant withdrawals/dropouts. If randomization and blinding were appropriate, 1 additional point was added for each. The total score ranged from 0 (bad) to 5 (good).

**Table 4:** NOS for cohort studies.

Study	Selection	Comparability	Outcome	Sum of score
Cho et al. [16]	●●●	●●	●●	7
Walline et al. [17]	●●●	●	●●	6
Kakita et al. [18]	●●●●	●●	●●●	9
Hiraoka et al. [20]	●●●●	●●	●●	8
Santodomingo-Rubido et al. [21, 24]	●●●●	●●	●●●	9
Chen et al. [23]	●●●●	●●	●●	8

NOS generates a quality score, maximum of 9 points, based on assessment of 3 study characteristics: patient selection methodology (maximum of 4 points), comparability of the study groups (maximum of 2 points), and outcomes measures (maximum of 3 points). The total score ranged from 0 (bad) to 9 (good).

**Table 5:** Results of sensitivity and subgroup analyses.

	Efficacy (AL change)			Acceptability (dropout rate)		
	Number of studies	Pooled WMD and 95% CI	Heterogeneity *p* value	Number of studies	Pooled OR and 95% CI	Heterogeneity *p* value
Standard analysis	8	−0.255 [−0.298, −0.212]	0.885	6	0.794 [0.516, 1.222]	0.320
Randomized	2	−0.282 [−0.379, −0.185]	0.664	2	1.400 [0.722, 2.713]	1.000
Nonrandomized	6	−0.248 [−0.296, −0.200]	0.788	4	0.510 [0.281, 0.923]	0.786
Prospective	6	−0.251 [−0.299, −0.203]	0.784	6	0.794 [0.516, 1.222]	0.320
Nested	2	−0.272 [−0.371, −0.172]	0.523			
Asian	6	−0.254 [−0.301, −0.207]	0.814	5	0.856 [0.546, 1.341]	0.330
Caucasian	2	−0.258 [−0.369, −0.148]	0.388	1	0.323 [0.060, 1.726]	1.000
A-Scan	2	−0.272 [−0.371, −0.172]	0.523			
IOLMaster	6	−0.251 [−0.299, −0.203]	0.784	6	0.794 [0.516, 1.222]	0.320
OK versus SV	5	−0.239 [−0.287, −0.191]	0.946	4	0.774 [0.442, 1.355]	0.303
OK versus SC	1	−0.320 [−0.499, −0.141]	1.000			
PRok versus SV	1	−0.320 [−0.517, −0.123]	1.000	1	0.794 [0.186, 1.302]	1.000
Tok versus SV	1	−0.330 [−0.485, −0.175]	1.000	1	1.400 [0.527, 3.718]	1.000

WMD = weighted mean difference; CI = credible intervals; AL = axial length; OR = odds ratio.

**Table 6:** Subgroup analyses of pooled myopic control rate of different treatment duration.

	Number of studies	Pooled myopic control rate
6 months	5	55%
12 months	7	51%
18 months	5	51%
24 months	8	41%

**Table 7:** Adverse events of included trials.

	Year	Adverse events
Cho et al. [16]	2005	Four subjects withdrawal because of corneal complications in OK group (2 with recurrent corneal staining and 2 with inflammation.)

Walline et al. [17]	2009	None of the dropouts were due to complications.

Kakita et al. [18]	2011	In the OK group, two patients had mild corneal erosion, which improved after 1 week of treatment cessation, and subsequent OK treatment was resumed without any sequelae. No other complications, such as corneal ulcer, were noted. There were no adverse events in the spectacle group.

Cho and Cheung [19]	2012	One recurrent corneal inflammation was reported in the control group and the subject was excluded from the study. Five ortho-k subjects were withdrawn from the study due to ocular health issue: three had mild rhinitis resulting in corneal staining, one had increased conjunctival hyperemia, and the remaining subject developed chalazion in the right eye. Ocular conditions and vision of these ortho-k subjects were not affected after cessation of ortho-k treatment.

Hiraoka et al. [20]	2012	Moderate superficial punctuate keratopathy was observed in 3 subjects and mild corneal erosion was found in 1 subject in the OK group, but these conditions were recovered completely after discontinuation of lens wear for 1 week. All subjects resumed OK treatment thereafter. No other severe complications, such as corneal ulcer, were noted in the OK group and there were no adverse events in the spectacle group.

Santodomingo-Rubido et al. [21, 24]	2012	Nine OK subjects showed adverse events (i.e., corneal staining, corneal abrasion, conjunctivitis, contact lens-induced peripheral ulcer, dimple veiling, blepharitis, and hordeolum). Two of them discontinued the study. The adverse events found with OK in this study are not considered to be serious, are similar to those reported with other contact lens types, and can be managed straightforwardly in clinical practice.

Charm and Cho [22]	2013	Corneal staining was observed in some subjects in both groups at each visit, but the incidence was generally higher in the PR ortho-k subjects. However, all stainings observed were not significant (all were grade 1) between the two groups of subjects who completed the study. Only one subject was withdrawn from the study due to grade 2 (coverage) peripheral corneal staining in OK group. No other adverse events were reported in either group of subjects.

Chen et al. [23]	2013	None of the dropouts in either group of subjects was due to ocular adverse events. Although ortho-k lens wear tended to increase the incidence of corneal staining in the peripheral cornea, the staining observed was considered to be mild as depth of staining was mostly superficial (Grade 1) and the average incidence was less than 10%.
